# Long-term neurocognitive and behavioral outcomes in survivors of pediatric brain tumors: a systematic review

**DOI:** 10.3389/fnins.2025.1587059

**Published:** 2025-05-30

**Authors:** Tirath Patel, Pulkit Johar, Vaishnavi Kanisetti, Sahithi Talacheru, Vadali Avinash, Arghadip Das, Sweta Sahu, Abhishek Goyal, Megan W. Szobody, Tori Sayers, Sriya Gullapalli, Murali M. Yallapu, Mushfiq H. Shaikh, Nikhilesh Anand, Kelsey Potter-Baker, Bharathi S. Gadad

**Affiliations:** ^1^Department of Neurosurgery, Trinity Medical Sciences University School of Medicine, Kingstown, Saint Vincent and the Grenadines; ^2^Department of Internal Medicine, AIIMS Rishikesh, Rishikesh, India; ^3^Department of Internal Medicine, Bhaskar Medical College, Hyderabad, India; ^4^Department of Internal Medicine, Mediciti Institute of Medical Sciences, Hyderabad, India; ^5^Department of Internal Medicine, Dr. Pinnamaneni Siddhartha Institute of Medical Sciences and Research Foundation, Vijayawada, India; ^6^Department of Internal Medicine, Nilratan Sircar Medical College and Hospital, Kolkata, India; ^7^Department of Internal Medicine, J.J.M. Medical College, Davanagere, Karnataka, India; ^8^Department of Neurology, JFK University Medical Center, Edison, NJ, United States; ^9^University of Texas Rio Grande Valley, School of Medicine, Edinburg, TX, United States

**Keywords:** neurocognition, behavior, pediatric tumor, systematic review, malignancy

## Abstract

**Background:**

Brain tumors are among the most common neoplasms in children. These patients suffer from neurocognitive impairment, treatment-related side effects, and experience a subpar quality of life (QoL), affecting academic endeavors, social interaction, and mental wellbeing.

**Methods:**

This review investigated different long-term neurocognitive and behavioral outcomes in pediatric brain tumor survivors and evaluated various effective treatment methods. We identified 75 relevant articles published between 2019 and 2024 using PubMed, PMC, Embase, and Google Scholar databases. Duplicates were removed, and 14 studies were finally selected following the PRISMA guidelines. Initial observations noted significant variations in the study methodologies and inclusion criteria.

**Results:**

Our study showed that children treated with proton radiotherapy experienced better neurocognitive and academic results than those treated with photon radiotherapy. Cognitive abilities were affected irrespective of the treatment, especially in early stage radiotherapy. Psychosocial impacts such as low self-esteem, depressive symptoms, and increased suicidal ideation were also demonstrated. Improvement in long-term outcomes was noted within therapeutic plans devoid of delayed high-dose radiotherapy and marrow ablation chemotherapy.

**Discussion:**

Increasing our understanding of the long-term effects associated with brain tumors using our current treatment methodology will help us formulate better treatment protocols and improve survivors’ quality of life.

## 1 Introduction

Pediatric brain tumors are abnormal growths of cells within the brain or central nervous system (CNS) that occur in children and adolescents (Lutz et al., 2022). These are the most common solid tumors in children and represent the leading cause of cancer-related mortality. On average, pediatric brain tumors represent approximately 20–30% of all childhood cancers worldwide (Thorbinson and Kilday, 2021). These tumors, which occur in approximately 1 in 2,000 children, have a major negative effect on the brain’s cognitive and psychological wellbeing. Annually, almost 4,000 children in the United States are diagnosed with brain or central nervous system tumors. Overall, the global incidence of pediatric brain tumors is estimated to be approximately 3–4 cases per 100,000 children per year, with variations depending on region, environmental factors, and genetic predispositions (Piñeros et al., 2016). Advances in diagnosis and treatment have significantly improved survival rates, with many children surviving into adulthood. However, long-term neurocognitive and behavioral outcomes in survivors remain a critical concern, as these tumors and their treatments can profoundly affect the developing brain.

Among several types of pediatric brain tumors (PBTs), medulloblastomas are the most common malignant brain tumors in children, typically arising in the cerebellum, which controls balance and coordination. These tumors can spread to other parts of the brain and spinal cord. Another common type of brain tumor, craniopharyngiomas, are benign tumors that develop near the pituitary gland, often causing hormonal imbalances and vision problems due to their location. Germ cell tumors originate from germ cells, usually found near the pineal gland or pituitary gland, and can be benign or malignant (Mastrangelo, 2023). The diagnosis of PBTs typically involves imaging studies, such as MRI or CT scans, along with biopsy procedures to determine the type and grade of the tumor. Treatment strategies often include a combination of surgery, radiotherapy, and chemotherapy. Key advancements include proton beam therapy, molecular-targeted therapies, and immunotherapy. Studies have shown that the timing and dosage of radiation therapy are critical, especially for young children. Exposure to high radiation doses can significantly lead to cognitive impairments. One study reported that approximately 40–80% of children who received craniospinal radiation experienced cognitive dysfunction (Duffner, 2004). Chemotherapy, although effective in treating certain tumors, can lead to long-term neurocognitive and endocrine effects. Endocrine sequelae affect 20–50% of childhood cancer survivors, depending on the chemotherapy regimen and radiation therapy involved (Gebauer et al., 2019). In infants, treatment regimens are more complicated, as the developing brain is particularly vulnerable to the toxic effects of treatments. Some PBTs are highly treatable with a good prognosis, while others, especially high-grade malignancies, are more challenging and have a poorer prognosis. The long-term effects of treatment, including potential neurocognitive and behavioral effects, are a significant consideration in managing these cases (Lassaletta et al., 2023). Recent developments in treatment protocols for pediatric brain tumors have focused on improving survival rates while minimizing long-term neurocognitive and developmental side effects (Pancaldi et al., 2023). Proton beam therapy is increasingly used in pediatric brain tumor treatment. It aims to minimize radiation exposure to healthy tissues, especially in sensitive areas such as the hippocampus, which is vital for memory. The introduction of targeted therapies, such as tyrosine kinase inhibitors (TKIs), is also promising for treating specific tumor types while limiting systemic toxicity. TKIs can reduce tumor size by up to 60% in certain pediatric brain tumors, offering an alternative to traditional therapies, such as chemotherapy, that have significant long-term effects (Zhou et al., 2022).

Several factors may influence the neurocognitive outcomes in these survivors, including tumor location, size, and histology, as well as the age at diagnosis, extent of surgical resection, and neurotoxicity of adjuvant therapies. Radiation therapy, particularly cranial irradiation at a young age, is a major contributor to cognitive decline. Due to their developing CNS, infants are particularly vulnerable to radiation therapy. Therefore, for this population, age-appropriate treatment protocols are essential, and alternative therapies, such as chemotherapy, may be preferred to limit exposure to radiation. Furthermore, emerging evidence suggests that genetic predispositions and molecular characteristics of the tumor may play a role in determining long-term outcomes (Robinson et al., 2010). Fortunately, over the past few decades, the five-year survival rate for pediatric patients diagnosed with brain tumors has reached an impressive rate of almost 75% (Hossain et al., 2021). Nevertheless, it remains a significant cause of concern, as the consequences of the tumor and its therapy can greatly influence cognitive and behavioral growth. Neurocognitive and behavioral impairments have a substantial impact on individuals’ academic performance, social interactions, and overall wellbeing, leading to major long-term consequences. The long-term effects faced by survivors can be grouped into the following categories: (1) neurocognitive late effects, (2) psychological and psychiatric late effects, and (3) social late effects. Late impacts, which include disturbances in cognition, education, attention, processing speed, executive functions, and memory, appear differently among survivors. Individuals may also experience behavioral disorders, including heightened anxiety, despair, and challenges in social interactions. Such enduring consequences can greatly impede their capacity to reach their highest potential and live satisfying lives (Mittal and Kent, 2017).

Typically, brain tumors and therapeutic interventions disrupt white matter pathways, resulting in a decrease in white matter and impaired neurocognitive functioning (Reeves et al., 2006). Survivors may experience neurological difficulties, including pain, seizures, loss of sensation, and visual impairments. These issues can have lasting effects on self-esteem and overall QoL (Mabbott et al., 2011). In addition, medical interventions may cause impaired hearing, which can be linked to poorer cognitive performance and display impairments in speech and language abilities. The impact of cranial radiation on younger children may also affect speech and language development, with delayed milestones observed in some cases (DeNunzio and Yock, 2020).

Those who have been treated for posterior fossa tumors may experience a persistent deterioration in working memory over time, even when their IQ levels remain consistent 20–40 years after diagnosis. Therefore, it is imperative to conduct a comprehensive evaluation to synthesize current data, pinpoint areas of knowledge deficiency, and provide guidance for future research and therapeutic practices (Riva and Giorgi, 2000). The neurocognitive effects, including memory and executive function deficits, are directly linked to the type and intensity of the treatment regimen, with radiation therapy contributing most significantly to these impairments (Wickborn et al., 2024).

Neurocognitive impairment significantly affects the psychological wellbeing of pediatric brain tumor survivors. Studies have shown that these individuals often experience psychiatric disorders, such as anxiety, depression, and post-traumatic stress disorder (PTSD), due to their experiences with cancer and treatment. Survivors may also face challenges related to emotional regulation and adjustment to life after treatment. The psychological burden can be further exacerbated by cognitive deficits, as difficulties in learning and memory affect academic success and social interactions, contributing to feelings of isolation and frustration (Söderström et al., 2022). These effects can significantly impact the survivor’s overall emotional health and ability to adapt to the demands of daily life.

The long-term social consequences of pediatric brain tumors are profound. Survivors may struggle with social integration and face difficulties in establishing and maintaining relationships, owing to both neurocognitive and psychological challenges. These difficulties are compounded by stigmatization and the challenges of reintegrating into school environments. Delays in developmental milestones, such as language and emotional regulation, can result in significant social withdrawal and reduced participation in age-appropriate activities. This further impacts the survivor’s ability to achieve a fulfilling social life (Cheung et al., 2019). Therefore, addressing these social aspects is crucial for improving the QoL of pediatric brain tumor survivors.

It is imperative to recognize that the late effects of pediatric brain tumors are not isolated to any single domain but intersect across these categories, influencing the survivor’s overall quality of life. Tailored interventions addressing each of these areas are critical for providing holistic care and improving long-term outcomes.

The following systematic review seeks to fill a significant void by consolidating the existing body of literature on neurocognitive and behavioral outcomes in children who have survived brain tumors. This review also aimed to analyze the outcomes. Through a methodical analysis of existing research, our objective is to offer a comprehensive summary of the present understanding and pinpoint crucial areas that necessitate additional exploration.

## 2 Methodology

### 2.1 Search strategy

Our systematic review followed the Preferred Reporting Items for Systematic Reviews and Meta-Analysis (PRISMA) guidelines (ref needed). We conducted a comprehensive search using the following MeSH terms: “Brain Neoplasms,” “Pediatrics,” “Cognition Disorders,” “Neurobehavioral Manifestations,” “Child,” “Survivors,” “Cognition,” “Behavior,” “Prognosis,” “Neurodevelopmental Disorders,” “Challenges,” and “Survivorship.” The search was performed using three databases: PubMed Central, Embase, and Google Scholar. We identified 75 articles using this search strategy. Our inclusion criteria were restricted to open access English-language studies focusing on pediatric patients (under 18 years of age) diagnosed with brain tumors, with a minimum follow-up period of 2 years post-treatment. We included studies that focused on long-term neurocognitive and behavioral outcomes in survivors of pediatric brain tumors and were published only in peer-reviewed journals between 2019 and 2024, as well as systematic reviews and meta-analyses.

### 2.2 Screening and selection

The screening and selection process involved a team of researchers to ensure rigorous evaluation and reduce bias. The initial screening of titles and abstracts was conducted independently by two reviewers. Studies that met the inclusion criteria were subjected to a full-text review. Any discrepancies between the reviewers during this process were resolved through discussion or consultation with a third reviewer. A PRISMA flowchart was used to illustrate the study selection process, detailing the number of records identified, screened, assessed for eligibility, and included in the review.

### 2.3 Study selection

**Figure 1:**
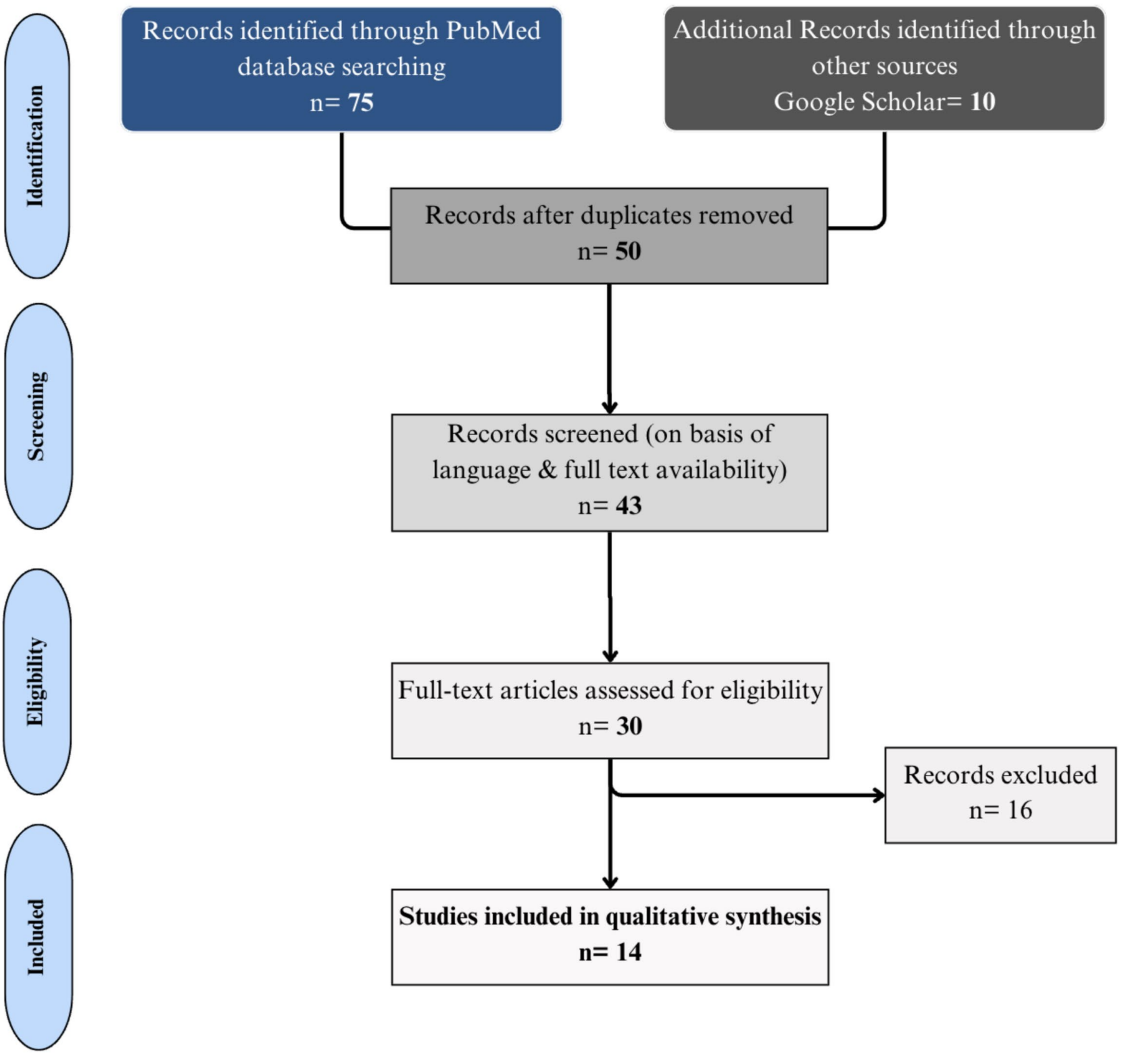
PRISMA diagram for studies selection, eligibility and data extraction.

Our initial search yielded 75 articles. After excluding duplicates, we screened the titles and abstracts of 68 unique records. Of the 68, 25 articles were excluded based on the inclusion criteria. The remaining 43 articles were assessed for eligibility, and finally, 14 articles were selected for review. The study selection process is described in detail in the PRISMA flowchart (Figure 1).

### 2.4 Data extraction and synthesis

Data extraction focused on key variables, such as study characteristics, patient demographics, tumor characteristics, and neurocognitive and behavioral outcomes. A summary of the extracted data is presented in Table 1.

**Table 1:** A summary of the key characteristics, patient demographics, tumor characteristics, and neurocognitive and behavioral outcomes of the 14 studies included in the data.

Study	Sample size	Subgroups	Age diagnosis/treatment	Gender (M/F)	Tumor location	Follow-up (years)	Follow-up age	FSIQ	Verbal comprehension & reasoning	Processing speed	Attention & working memory	Verbal learning	Outcome
Child et al. (2021) [23]	88	Focal = 43, CSI = 45	6.0	58/30	Supratentorial = (Focal = 23, CSI = 16) Infratentorial = (Focal = 19, CSI = 28)	1 year	4 years	Focal = 94 CSI = 77.5	VCI = (Focal = 99.5, CSI = 84.3)	PSI = (Focal = 82.95, CSI = 72.8)	WMI = (Focal = 97, CSI = 81.9)	Focal = 97, CSI = 85.4	The study revealed that individuals who have survived pediatric brain tumors are most susceptible to cognitive and scholastic challenges, with PRT CSI presenting a lower level of risk in comparison to XRT CSI. The performance of individuals in the focal PRT group, which is suitable for their age, suggests positive overall results.
Helligsoe et al. (2023)	161	Focal = 29, WBI = 30, No treatment = 102	9.1	74/87	Cerebellum = 69 Cerebrum = 32 Supratentorial central area = 12 Hypothalamus or pituitary region = 18 Brain stem = 12 Optic nerve or chiasma = 10 Pineal gland = 8	5 years	24.3		−1.03	−1.43	−0.26	Not mentioned	Childhood brain tumor survivors frequently encounter neurocognitive deficits, diminished quality of life, and a significant burden of symptoms, but these problems may not be directly linked.
Tonning Olsson et al. (2024)	151	Not mentioned	8.4	88/63	Ependymomas and choroid plexus tumors = 13 Astrocytomas = 64 Intracranial and intraspinal embryonal tumors = 21 Other gliomas = 13 Other specified intracranial neoplasms = 35 Germ cell tumors = 1	3 years	Not mentioned	Not mentioned	VRI = 89.1	CPS = 79.7	WMI = 78.3	VLI = 102.1	The study demonstrates that individuals who have survived primary brain tumors (PBTs) are more likely to experience substantial decreases in neurocognitive scores, independent of the type of therapy they received. Lower cognitive function is connected with male sex and tumor placement, although there is no evidence of faster reductions. Survivors who underwent whole-brain radiation therapy (WBRT), targeted cranial radiation therapy (CRT), chemotherapy, surgery, or a ventriculoperitoneal (VP) shunt exhibited the most rapid decline.
Levitch et al. (2021)	51	Not mentioned	6.41 years	Not mentioned	Supratentorial = 21 Infratentorial = 30	6–10 years	11.1 years	87.9	Verbal IQ = 19	Not mentioned	Not mentioned	Not mentioned	Long-Term Factors (LTF) revealed that young survivors of pediatric brain tumors in high school II demonstrated consistent intellectual performance and experienced less long-term consequences. However, a significant proportion of youngsters (20–33%) experienced below-normal levels, particularly those who were treated with HD-MTX, which put them at a greater risk.
Eaton et al. (2020)	59	Not mentioned	2.5, PRT	15/25	Ependymoma = 22 Medulloblastoma = 7	6.7 years	9.1 years	Not mentioned	Not mentioned	Not mentioned	Not mentioned	Not mentioned	The study demonstrates that the quality of life (QoL) of young children who get Prophylactic Therapy (PRT) for brain tumors varies greatly and is greatly affected by the severity of neurological damage at the time of diagnosis and treatment. Nevertheless, more than one-third of patients indicate comparable quality of life scores to that of healthy youngsters.
Söderström et al. (2022)	50	Not mentioned	9.50y, radiotherapy	29/21	Embryonal = 15 Astrocytic = 9 Ependymal = 10 Craniopharyngioma = 6 Pituitary adenoma = 1 Pineoblastoma = 1 Germ cells tumors = 6	5 years	10.34	91.06	VRI = 94.35 PRI = 99.0	PSI = 84.17	WMI = 89.44	Not mentioned	Prior to radiation therapy (RT), neurocognitive abilities were impacted, with these effects becoming more noticeable as time progressed. Specifically, working memory and processing speed were particularly impaired.
Roth et al. (2020)	70	Focal = 33 Craniospinal = 37	7.50, proton radiotherapy	23/14	Supratentorial = 34 Infratentorial = 35	5 years	12.84	CSI = 85.30 Focal = 97.76	VCI (CSI = 88.79, Focal = 99.42) PRI (CSI = 90.88, Focal = 10.3.67)	PSI (CSI = 78.03, Focal = 87.45)	WMI = (CSI = 87.94, Focal = 97.60)	Not mentioned	Survivors of Focal PRT exhibited predominantly favorable results, however they displayed deficiencies in processing speed and certain elements of adaptive functioning. Exposure to CSI was linked to persistently negative cognitive and adaptive results. The heightened susceptibility to adaptive dysfunction in the PRT CSI group can be attributed to the impact of CSI on cognition. It is nevertheless crucial to make efforts to decrease the amount of tissue that is exposed to radiation therapy (RT).
Kahalley et al. (2019)	93	Proton SCI = 31, Proton focal = 31, surgery = 40	9.7y, PRT	48/45	Supratentorial = 57 Infratentorial = 36	3 years	Not mentioned	Proton SCI = 94.2, Proton focal = 95.9, surgery = 93.2	CSI = (Proton SCI = 96.3, Proton focal = 98.0, surgery = 93.6) PRI = (Proton SCI = 98.3, Proton focal = 96.7, surgery = 96.3)	Not mentioned	WMI = (Proton SCI = 95.8, Proton focal = 100.9, surgery = 92.5)	Not mentioned	Survivors who underwent Focal PRT experienced consistent neurocognitive functioning throughout their recovery. The results were comparable regardless of whether patients got focused PRT or no radiotherapy, even in neurocognitive areas that are known to be more vulnerable to radiation. The Proton CSI has been identified as a neurocognitive risk factor, which aligns with the findings of research on photon consequences.
Sharkey et al. (2022)	166	Brain tumor = 96, acute lymphoblastic leukemia = 52, others = 18	6.21	90/76	Not mentioned	5.32 years	11.57	Not mentioned	Not mentioned	Not mentioned	66.19	Not mentioned	A significant proportion of adolescents undergoing cancer treatment suffer from somatic symptoms and associated neurocognitive difficulties. There is a need to screen for suicidal ideation (SI) and conduct a more thorough evaluation of the relationship between executive functioning and SI in children with cancer.
Alias et al. (2020)	114	Brain tumor = 38 Leukemia = 38 Healthy control = 38	7.2	72/42	Supratentorial = 21 Infratentorial = 17		12.5 years	Not mentioned	Not mentioned	Not mentioned	Not mentioned	Not mentioned	The childhood brain tumor survivors at our facility exhibited deficiencies in social skills and attention, underscoring the necessity for tailored psychological support treatments after treatment.
Willard et al. (2021)	98	Not mentioned	1.33	50/48	Not mentioned	Not mentioned	10.86	Not mentioned	Not mentioned	Not mentioned	Not mentioned	Not mentioned	The study demonstrates that the functioning of retinoblastoma patients improves by the age of 10. However, there are early decreases and challenges associated with enucleation-only treatment, which highlights the necessity for early intervention programs, ongoing monitoring, and heightened awareness of risk factors.
Seck et al. (2022)		PBTS-SgM = 7, PBTS-Sg + AM = 6, HC = 10	7.5	15/32	Infratentorial = 6 Supratentorial = 6 Midbrain Tectal = 1	6 years	12.50	Not mentioned	Not mentioned	PSI Composite score = (PBTS SgM = 90.29, PBTS-Sg + AM = 91.33, HCM = 104.50)	Not mentioned	Not mentioned	The study revealed that increased connectivity in the (SN) and (DMN) in individuals with Posterior Brain Trauma Syndrome (PBTS) was linked to enhanced task performance and executive abilities as judged by parents. Conversely, heightened connectivity in the Central Executive Network (CEN) was related with lower executive skills.
Srsich et al. (2024)	161	Not mentioned	6.83	92/69	Supratentorial = 80 Infratentorial = 75	5 years	13.4 years	Not mentioned	VR = 94.95 PRI = 95.55	PSI = 92	Not mentioned	Not mentioned	The study cognitive abilities related to information processing and speed, but PNORTI does not show a correlation with neuropsychological outcomes. Subsequent investigations should determine the specific threshold scores that indicate the danger level for survivors in a clinical setting.
Cheung et al. (2019)	157	Brain tumor = 77, other tumors = 80	11.77y	89/68	Not mentioned	5 years	Not mentioned	Not mentioned	Not mentioned	Not mentioned	Not mentioned	Not mentioned	This study demonstrates that pediatric brain tumor survivors exhibit inferior psychological wellbeing, characterized by a higher prevalence of depressive symptoms, lower levels of self-esteem, and a degraded quality of life in comparison to other pediatric cancer survivors. It highlights the importance for healthcare practitioners to create and assess therapies aimed at improving the psychological wellbeing and quality of life of these survivors.

PRI, Perceptual Reasoning Index; VRI, Verbal Reasoning Index; WMI, Working Memory Index; VCI, Verbal Comprehension Index; CPS, Cognitive processing speed; VL, Verbal Learning; PRT, Proton Radiotherapy.

## 3 Results and discussion

This systematic review aimed to assess long-term neurocognitive and behavioral outcomes in survivors of pediatric brain tumors (PBTs). Given the significant cognitive and psychosocial challenges these survivors experience, understanding their outcomes is crucial for improving their QoL. The review synthesized data from 14 studies, each examining different aspects of neurocognitive and neuropsychiatric outcomes among pediatric brain tumor survivors. The included studies varied in patient demographics, tumor types, and treatment modalities, providing a comprehensive overview of the current research landscape.

The neurocognitive and neuropsychiatric challenges faced by PBT survivors are significant. Survivors often experience deficits in the intelligence quotient (IQ), processing speed, working memory, and academic performance. Several studies have also pointed to the long-term implications of these deficits on educational and occupational functioning (Alias et al., 2020; Child et al., 2021; Roth et al., 2020). Neurocognitive impairments have been commonly reported across studies, affecting various domains, such as intelligence, verbal comprehension, processing speed, attention, memory, learning, and executive functioning. Child et al. (2021) found that children treated with proton radiotherapy (PRT) generally showed good neurocognitive and academic long-term effects compared to those who received photon radiotherapy (XRT) (Child et al., 2021). However, even in PRT recipients, the greatest damage was noted after craniospinal irradiation (CSI). Helligsoe et al. (2023) and Tonning Olsson et al. (2024) emphasized the elevated risk of neurocognitive decline among brain tumor survivors, with impairments in attention, memory, and executive functioning being prevalent (Helligsoe et al., 2023; Tonning Olsson et al., 2024). A study showed that childhood brain tumor survivors, regardless of treatment type (focal, whole-brain irradiation, or no treatment), experienced significant neurocognitive impairment, with reduced QoL being a common outcome (Helligsoe et al., 2023).

Levitch et al. (2021) reported average intellectual functioning in children treated with high dosages but noted below-average performance in the receptive language and mathematics cognitive domains, suggesting that while some cognitive abilities might be preserved, others are significantly affected by intensive treatments (Levitch et al., 2021). Eaton et al. (2020) and Roth et al. (2020) corroborated cognitive impairment in specific abilities, highlighting that early stage radiotherapy could act as a confounding factor for cognitive abilities, particularly in younger children (Eaton et al., 2020; Roth et al., 2020). QoL assessments revealed that neurocognitive impairments significantly affected the overall wellbeing of the survivors. Söderström et al. (2022) noted that radiotherapy at an early age could confound the reduction in cognitive abilities, which in turn, affected the QoL of survivor (Söderström et al., 2022). A study on PRT highlighted cognitive dysfunction as a notable post-treatment outcome that could contribute to a decline in QoL (Roth et al., 2020).

A significant advancement in the treatment of pediatric low-grade glioma (pLGG) is the use of MEK inhibitors. MEK inhibitors, such as trametinib, are becoming a standard treatment option for pLGG, particularly in cases where these tumors are refractory to conventional therapies, such as radiotherapy. MEK inhibitors function by targeting the MAPK/ERK pathway, which is involved in tumor growth and survival. However, recent studies have suggested that while MEK inhibitors show promise in controlling tumor progression, they may also have significant neurocognitive effects. Research indicates that patients treated with MEK inhibitors can experience cognitive impairments, particularly in areas such as processing speed, attention, and working memory (Sait et al., 2023; Selt et al., 2020).

These effects, although less well understood than those caused by radiation therapy, point to the importance of closely monitoring neurocognitive functioning in children undergoing MEK inhibitor treatment. While MEK inhibitors may not induce the same degree of widespread neurotoxicity as radiotherapy, their impact on specific cognitive domains could still be significant, particularly given the age and developmental stage of the pediatric population (Walsh et al., 2021). Future research should aim to compare the neurocognitive outcomes of MEK inhibitors and radiotherapy directly to better understand the comparative risks of these treatment modalities.

Willard et al. (2021) and Alias et al. (2020) explored QoL in broader patient groups and specific tumor types, respectively. Willard’s findings indicated that a subset of patients showed significant cognitive and/or psychosocial effects, reinforcing the notion that neurocognitive outcomes directly influence QoL (Willard et al., 2021; Alias et al., 2020). Different treatment modalities, including surgery, chemotherapy, and radiotherapy (both photon and proton), were examined for their impact on neurocognitive functioning and QoL. The study by Child et al. (2021), Kahalley et al. (2019), and Seck et al. (2022) indicated that proton radiotherapy (PRT), especially focal PRT, was associated with relatively stable neurocognitive functioning in survivorship (Child et al., 2021; Kahalley et al., 2019; Seck et al., 2022). However, proton CSI has emerged as a neurocognitive risk factor, demonstrating the need for careful selection and application of radiotherapy techniques. Sharkey et al. (2022) examined the broader psychological impacts of treatments, such as suicidal ideation and overall mental health, underscoring the necessity of comprehensive psychological assessments and interventions for brain tumor survivors (Sharkey et al., 2022). Levitch et al. (2021) and Srsich et al. (2024) highlighted the variability in individual outcomes and the potential benefits of treatment strategies that avoid or delay radiotherapy by using high-dose, marrow-ablative chemotherapy and autologous hematopoietic cell transplantation (AuHCT) (Levitch et al., 2021; Srsich et al., 2024). These strategies might decrease neurocognitive and social–emotional decline in young pediatric brain tumor survivors, suggesting alternative approaches to improve long-term outcomes.

Cheung et al. (2019) addressed the critical psychological and physical outcomes faced by pediatric brain tumor survivors (PBTS) compared to survivors of other pediatric cancers (Cheung et al., 2019; Mittal and Kent, 2017). Conducted on 157 pediatric cancer survivors aged 8–16 years, it included 77 PBTS and 80 survivors of other cancers. This study aimed to assess the impact of cancer and its treatment on physical and psychological wellbeing and QoL (Cheung et al., 2019). The findings revealed that more than 70% of PBTS patients exhibited significant depressive symptoms (Cheung et al., 2019). These survivors reported lower self-esteem and compromised QoL than survivors of other pediatric cancers. Research has shown a clear link between the severity of depressive symptoms and lower levels of self-esteem and QoL (Cheung et al., 2019).

This review contributes to the growing body of research in cancer neuroscience, particularly in the domain of pediatric neurooncology. Knowledge about neurocognitive and behavioral outcomes in pediatric brain tumor survivors not only informs clinical practice but also enriches the broader context of cancer neuroscience. These findings highlight the profound impact of tumor location, treatment modality, and age at diagnosis on neurocognitive development, aligning with the broader goal of understanding how cancer treatment affects the brain. By exploring the cognitive consequences of various treatment modalities, including the emerging use of MEK inhibitors, this review extends our understanding of how targeted therapies and conventional treatments influence neuroplasticity, memory, and learning in the pediatric population. Improving the long-term wellbeing of these survivors requires a comprehensive approach that integrates both cognitive and behavioral health strategies. This review contributes valuable insights to the field, underscoring the importance of addressing these long-term effects and providing a foundation for future research aimed at enhancing the QoL of pediatric brain tumor survivors.

### 3.1 Limitations of the review

There are a few methodological limitations, one of which is sample size, limiting generalizability and statistical power. Furthermore, the selected studies varied significantly in design, including retrospective and prospective analyses, longitudinal follow-up, and cross-sectional assessments. These differences pose challenges in synthesizing the results and drawing definitive conclusions. There is also notable variability in treatment protocols, treatment regimens, and assessment tools used, contributing to outcome heterogeneity and limiting the robustness of the findings. Future research should address these limitations using standardized methodologies and incorporating a wider range of studies.

### 3.2 Future implications and recommendations

Regular follow-up until the age of 15 years is crucial for the early detection and management of neurocognitive and behavioral issues in PBT survivors. These patients are at risk for cognitive impairment, and timely interventions can significantly improve outcomes. Tailored follow-up schedules based on individual risk factors, such as tumor location and treatment type, are essential. While family counseling provides emotional support, educating families about long-term effects and management strategies enhances survivors’ home environments. Early personalized rehabilitation is key to improving cognitive and functional outcomes and helping survivors reintegrate into daily life and achieve long-term success.

Future research should focus on longitudinal studies with larger and more diverse cohorts to better understand the trajectory of neurocognitive and neuropsychiatric outcomes. Methodological improvements, such as standardized assessment tools and protocols, will enhance the credibility of future studies. Investigating potential interventions, such as cognitive training programs and psychosocial support, can provide insights into improving outcomes. Research should also explore the mechanisms underlying these deficits in order to develop targeted therapies.

## 4 Conclusion

Despite advancements in treatment modalities, such as proton radiotherapy (PRT) and photon radiotherapy (XRT), PBT survivors continue to face significant cognitive and neuropsychiatric challenges that adversely affect their quality of life. PRT and XRT provide some neurocognitive advantages and are associated with considerable risks, including cognitive impairment and diminished quality of life. Personalized monitoring and follow-up schedules, family counseling, and tailored rehabilitation programs are crucial for mitigating developmental delays, providing psychological support to improve cognitive outcomes, and enhancing overall wellbeing. Future research should focus on longitudinal studies with larger, more diverse populations and standardized protocols to better understand neurocognitive and neuropsychiatric trajectories.

## Data Availability

The original contributions presented in the study are included in the article/supplementary material, further inquiries can be directed to the corresponding author.
